# Genome-wide identification and expression profile under abiotic stress of the barley non-specific lipid transfer protein gene family and its Qingke Orthologues

**DOI:** 10.1186/s12864-021-07958-8

**Published:** 2021-09-20

**Authors:** Jiecuo Duo, Huiyan Xiong, Xiongxiong Wu, Yuan Li, Jianping Si, Chao Zhang, Ruijun Duan

**Affiliations:** 1 College of Eco-Environmental Engineering, Qinghai University, Xining, 810016 Qinghai Province China; 2 Qinghai Qaidam Vocational & Technical College, Delingha, 817000 Qinghai Province China; 3 College of Agriculture and Animal Husbandry, Qinghai University, Xining, 810016 Qinghai Province China

**Keywords:** Barley, Qingke, Non-specific lipid transfer proteins, Gene family, Expression profile, Abiotic stress

## Abstract

**Background:**

Plant non-specific lipid transfer proteins (nsLTPs), a group of small, basic ubiquitous proteins to participate in lipid transfer, cuticle formation and stress response, are involved in the regulation of plant growth and development. To date, although the nsLTP gene family of barley (*Hordeum vulgare* L.) has been preliminarily identified, it is still unclear in the recently completed genome database of barley and Qingke, and its transcriptional profiling under abiotic stress has not been elucidated as well.

**Results:**

We identified 40 barley nsLTP (*HvLTP)* genes through a strict screening strategy based on the latest barley genome and 35 Qingke nsLTP (*HtLTP*) orthologues using blastp, and these *LTP* genes were divided into four types (1, 2, D and G). At the same time, a comprehensive analysis of the physical and chemical characteristics, homology alignment, conserved motifs, gene structure and evolution of HvLTPs and HtLTPs further supported their similar nsLTP characteristics and classification. The genomic location of *HvLTPs* and *HtLTPs* showed that these genes were unevenly distributed, and obvious *HvLTP* and *HtLTP* gene clusters were found on the 7 chromosomes including six pairs of tandem repeats and one pair of segment repeats in the barley genome, indicating that these genes may be co-evolutionary and co-regulated. A spatial expression analysis showed that most *HvLTPs* and *HtLTPs* had different tissue-specific expression patterns. Moreover, the upstream cis-element analysis of *HvLTPs* and *HtLTPs* showed that there were many different stress-related transcriptional regulatory elements, and the expression pattern of *HvLTPs* and *HtLTPs* under abiotic stress also indicated that numerous *HvLTP* and *HtLTP* genes were related to the abiotic stress response. Taken together, these results may be due to the differences in promoters rather than by genes themselves resulting in different expression patterns under abiotic stress.

**Conclusion:**

Due to a stringent screening and comprehensive analysis of the *nsLTP* gene family in barley and Qingke and its expression profile under abiotic stress, this study can be considered a useful source for the future studies of *nsLTP* genes in either barley or Qingke or for comparisons of different plant species.

**Supplementary Information:**

The online version contains supplementary material available at 10.1186/s12864-021-07958-8.

## Key message

• 40 barley nsLTP (*HvLTP)* and 35 Qingke Orthologues nsLTP (*HtLTP*) genes were identified through stringent screening using the new barley and Qingke genomes and divided into four types (1, 2, D and G) by comprehensive analysis.

• The *HvLTPs* and *HtLTPs* were unevenly distributed in barley and Qingke chromosomes and had obvious gene clusters.

• Combined with the analysis of upstream cis-elements of *HvLTPs* and *HtLTP*s and their expression pattern under abiotic stress, it was found that numerous *HvLTP* and *HtLTP* genes may change their regulatory modes due to different upstream cis-elements and cause different abiotic stress responses.

## Background

Plant lipid transfer protein (LTPs), named for their function that transfer phospholipids and fatty acids between cell membranes in vitro, and they are also known as non-specific LTPs (nsLTPs) because of the characteristic of non-specific binding to different lipids [1, 2]. Plant nsLTPs are usually 6.5 to 10.5 kDa, although some nsLTPs are approximately 15 kDa, and their isoelectric points (pIs) are usually between 8.5 and 12 (occasionally less than 5). Therefore, nsLTPs are a group of small, basic and ubiquitous proteins, that have eight cysteine residue motifs (8CM, ECM) in a highly conserved backbone sequence, C-Xn-C-Xn-CC-Xn-CXCXn-C-Xn-C, and a high content of α-helices with a central hydrophobic cavity to bind lipids. Almost all nsLTPs carry an N-terminal signal peptide in their nascent polypeptides [3–5]. Plant nsLTPs are involved in multiple physiological functions, such as cuticular lipid transport, cutin synthesis, cell wall extension, pollen development, pollen tube growth and guidance, stigma and pollen adhesion, plant signalling, and seed maturation [1, 6–8]. In addition, some plant nsLTPs have been identified as related allergens in plant food and pollen [9–11]. The expression of some nsLTPs can be induced by biotic and abiotic stresses, including low or high temperature, drought, heavy metal exposure and disease [12–18]. In particular, many studies have shown that nsLTP genes are closely related to abiotic stress resistance in plant [9, 12, 13, 19, 20].

Barley (*Hordeum vulgare* L.) is the fourth largest crop in the world after wheat, rice, and maize, and it is one of the oldest food and feed crop in the world (http://faostat.fao.org). It is an important crop in China that has high economic and food value, especially on the Qinghai-Tibet Plateau. Tibetan hulless barley (highland barley), called “Qingke” in Chinese and “nas” in Tibetan, is the principal cereal cultivated on the Qinghai-Tibetan Plateau for at least 3500 years. Due to its high soluble dietary *β*-glucan and arabinoxylan contents, it is beneficial to human health and has attracted considerable interest [21–23]. Karen Skriver cloned and analysed the gene structure of the LTP1 gene in barley [24]. The crystal structure of this gene, specifically in ligand binding preferences, was also studied [25]. Then, some other barley genes (*LTP2*, *LTP3*, and *LTP4*) have been cloned in succession [26]. Interestingly, the barley nsLTP gene (*blt4*) was also found in the cold stress response [27, 28]. The latest physical, genetic and functional sequence assemblies of the barley and Qingke genomes were completed in 2012 and 2016 [29, 30] and 2015, 2018, and 2020 [22, 31, 32], respectively, and they provided an important reference for future crop breeding, improvement, gene function and evolution research.

Barley has strong drought resistance, low-temperature tolerance, and salt and alkali capacity, and its environmental adaptation mechanism is complex [19, 20]. Qingke is mainly cultivated on the Tibetan Plateau (>4000 m above sea level), which has high UV-B radiation, low temperatures and low barometric pressure, and thus may therefore have greater extreme environmental adaptability than cultivated barley from other regions [21, 31]. Currently, the nsLTP genes and their multigene families have been studied in many plants, including Arabidopsis, carrot, rape (*Brassica napus*), broccoli (*Brassica oleracea*), tobacco (*Nicotiana tabacum*), sugar beet (*Beta vulgaris*), sesame (*Sesamum indicum*), tomato (*Solanum lycopersicum*), maize (*Zea mays*), sorghum (*Sorghum vulgare)*, cotton (*Gossypium hirsutum*), rice (*Oryza sativa*) and wheat (*Triticum aestivum*) [1, 4, 9, 13, 17, 33–39]. For barley, although 70 barley nsLTP genes have been identified in 2012 barley genome [40], there are still many deficiencies that need further comprehensive research by using the genome database of barley recently completed in 2016. At the same time, the aim is to further understand the molecular mechanism underlying the stronger ability of Qingke to adapt to extreme environments than barley. Therefore, in this study, nsLTP gene family members were identified and a bioinformatics analysis of barley and Qingke was performed through strict screening based on the latest barley and Qingke genomes. Using qRT-PCR technology, the expression profile of this gene family under abiotic stress was also discussed. The results of this study lay a foundation for further studies on the biological and molecular functions of nsLTP genes in barley and Qingke.

## Results

### Identification, sequence analysis and classification of HvLTPs and HtLTPs

**Fig. 1:**
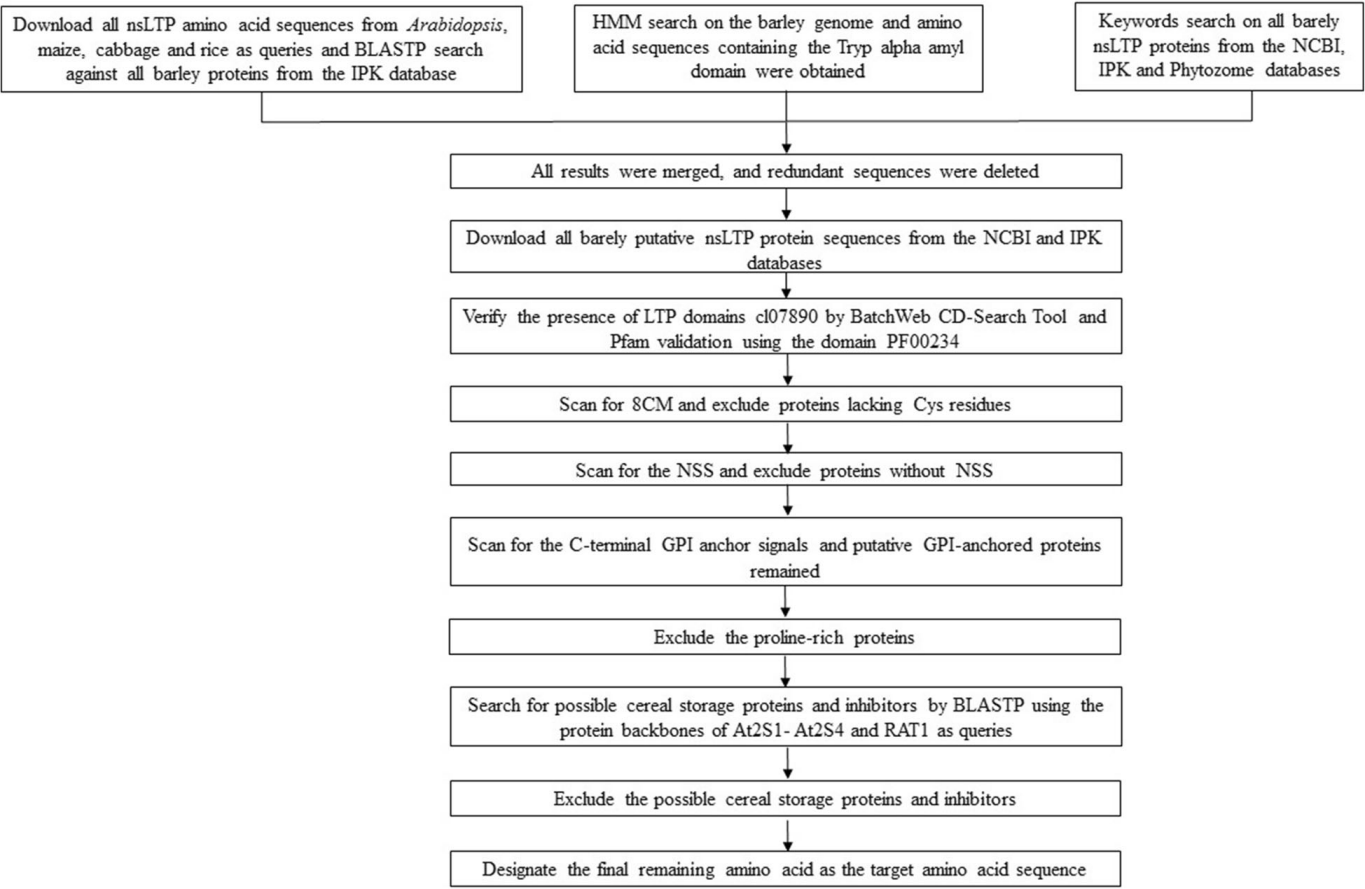
Workflow of HvLTP identification and data mining

To identify the entire collection of putative non-redundant nsLTP genes in the barley genome, an accurate search workflow of nsLTP identification and data mining was performed (Fig. 1). Initially, three search methods were used to identify barley nsLTP family members. First, 109 amino acid sequences were obtained after conducting a BLASTP analysis of the IPK Barley BLAST Server using previously reported nsLTP protein sequences of Arabidopsis (79), maize (63), cabbage (63) and rice (77) as queries (Table S1), and the redundancy was checked. Second, a total of 265 proteins with the conserved Tryp alpha amyl domain (Pfam domain PF00234) were retrieved by the HMM search method. Third, the 39 relevant barley nsLTP genes were downloaded by keyword searches from the NCBI, IPK and Phytozome databases. Then, the above results were merged, and the redundant sequences were examined and removed. Finally, all the barley deduced nsLTP protein sequences were downloaded from the NCBI and IPK databases and the presence of LTP domain cl07890 was verified by BatchWeb CD-Search and Pfam validation using the domain PF00234. After that initial identification step, a total of 160 putative nsLTP protein sequences were identified. Then, each of the deduced protein sequences was manually assessed through the analysis of the cysteine residue motifs (8CM), and 107 proteins lacking the Cys residues were omitted from the remaining set. In addition, 11 proteins lacking N-terminal signal sequences (NSS) were also excluded by NSS prediction, and 8 proteins with C-terminal glycosylphosphatidylinositol (GPI) anchors remained by GPI anchor signal prediction. Subsequently, 2 proline-rich proteins were also excluded, and no proteins similar to At2S1- At2S4 and RATI were found. Finally, we identified 40 nsLTPs in the whole barley genome and named them HvLTPs, and 35 nsLTPs were also identified in Qingke genome by local BLAST analysis with HvLTPs and named them HtLTPs (Table 1). Furthermore, to better understand the characteristics of the HvLTP and HtLTP proteins, we analysed the theoretical isoelectric point (pI) and molecular weight (MW) for all putative HvLTP and HtLTP proteins and summarized them in Table 1. As shown in Table 1, considering the mature form of nsLTPs, the average length is 132 aa (91–200 aa), with a molecular mass ranging from 9206.81 to 19,981.15 Da. The average Mw of HvLTPs is 13,344 Da, and the average theoretical pI of HvLTPs is 8.07, while the average Mw of HtLTPs is 13,238 Da and average theoretical pI of HtLTPs is 7.94, which demonstrates its small basic properties with subtle differences. We also analysed the instability index, aliphatic index and grand average of hydropathicity (GRAVY) of the HvLTPs and HtLTPs. The results showed that most instability index of HvLTPs and HtLTPs were greater than 40, the most aliphatic indices were greater than 75.5 and the GRAVY value was greater than zero, which indicated that most HvLTPs and HtLTPs were unstable, aliphatic and hydrophobic proteins.

**Table 1:** Characteristics of the different types of non-specific lipid transfer proteins found in barley (HvLTPs) and Qingke (HtLTPs)

Gene name	barley gene ID	Qingke gene ID	Chromosome	Amino acids		pI		Mass (Da)		Instability index		Aliphatic index		Grand average of hydropathicity (GRAVY)	
				Barley	Qingke	Barley	Qingke	Barley	Qingke	Barley	Qingke	Barley	Qingke	Barley	Qingke
Type1															
HvLTP1.1	HORVU2Hr1G107470.2		2	118		8.89		11,560.52		23.32		85.42		0.613	
HvLTP1.2/HtLTP1.2	HORVU2Hr1G107480.1	KAE8807755.1	2	120	120	9.03	9.03	11,856.79	11,856.79	31.67	31.67	84.75	84.75	0.55	0.55
HvLTP1.3/HtLTP1.3	HORVU2Hr1G107460.1	KAE8775919.1	2	125	125	9.82	9.82	12,233.34	12,233.34	38.53	38.53	98.8	98.8	0.51	0.51
HvLTP1.4	HORVU3Hr1G009560.1		3	115		9.46		11,221.08		25.46		89.39		0.487	0.487
HvLTP1.5/HtLTP1.5	HORVU3Hr1G009510.1	KAE8776966.1	3	126	126	4.35	4.35	12,730.43	12,730.43	61.65	61.65	82.22	82.22	0.213	0.213
HvLTP1.6/HtLTP1.6	HORVU3Hr1G009370.5	KAE8788659.1	3	115	115	9.32	9.32	11,099.06	11,099.06	33.71	33.71	95.39	95.39	0.608	
HvLTP1.7/HtLTP1.7	HORVU3Hr1G009490.4	KAE8788658.1	3	115	115	9.32	9.32	11,175.17	11,175.17	34.44	34.44	92.78	92.78	0.59	0.59
HvLTP1.8/HtLTP1.8	HORVU3Hr1G009520.2	KAE8788661.1	3	115	115	9.88	9.88	11,410.5	11,410.5	40	40	93.65	93.65	0.461	0.461
HvLTP1.9	HORVU3Hr1G009360.8		3	168	168	8.97		16,215.03		34.24		106.07		0.749	
HvLTP1.10/HtLTP1.10	HORVU3Hr1G029430.1	KAE8792517.1	3	155	155	8.65	8.65	16,185	16,185	28.35	28.35	89.61	89.61	0.61	0.61
HvLTP1.11/HtLTP1.11	HORVU4Hr1G022780.2	KAE8800812.1	4	111	111	8.7	8.7	10,982.81	10,982.81	49.05	49.05	85.41	85.41	0.34	0.34
HvLTP1.12/HtLTP1.12	HORVU4Hr1G022770.1	KAE8800813.1	4	120	120	9.41	9.41	11,746.77	11,746.77	42.01	42.01	93.75	93.75	0.631	0.631
HvLTP1.13/HtLTP1.13	HORVU5Hr1G046550.1	KAE8791291.1	5	117	117	8.7	8.7	12,297.24	12,297.24	35.23	35.23	95.04	95.04	0.087	0.087
HvLTP1.14/HtLTP1.14	HORVU5Hr1G046520.1	KAE8791289.1	5	149	149	9.58	9.58	15,377.09	15,377.09	45.4	45.4	87.85	87.85	0.316	0.316
HvLTP1.15/HtLTP1.15	HORVU7Hr1G080360.2	KAE8796122.1	7	120	120	3.74	3.74	12,209.7	12,209.7	63.06	63.06	86.5	86.5	0.307	0.307
HvLTP1.16/HtLTP1.16	HORVU5Hr1G111050.1	KAE8788807.1	5	128	128	8.51	8.51	12,881.01	12,881.01	49.5	49.5	88.67	88.67	0.403	0.403
Type2															
HvLTP2.1/HtLTP2.1	HORVU1Hr1G083160.1	KAE8821628.1	1	91	91	9.27	9.27	9206.81	9206.81	27.9	27.9	74.29	74.29	0.448	0.448
HvLTP2.2/HtLTP2.2	HORVU1Hr1G083170.1	KAE8802268.1	1	94	94	8.93	8.93	9370.08	9370.08	41.66	41.66	92.66	92.66	0.511	0.511
HvLTP2.3/HtLTP2.3	HORVU2Hr1G108660.1	KAE8821633.1	2	95	95	7.48	7.48	9602.32	9602.32	47.09	47.09	94.74	94.74	0.549	0.549
HvLTP2.4/HtLTP2.4	HORVU4Hr1G089490.2	KAE8784379.1	4	91	91	8.72	8.72	9439.32	9439.32	37.88	37.88	92.42	92.42	0.502	0.502
HvLTP2.5/HtLTP2.5	HORVU4Hr1G089500.1	KAE8777687.1	4	97	97	9.72	9.72	10,393.17	10,393.17	66.3	66.3	79.69	79.69	0.026	0.026
Type D															
HvLTPd1	HORVU1Hr1G043430.1		1	139		9		14,388.36		56.85		73.02		0.168	
HvLTPd2/HtLTPd2	HORVU2Hr1G102110.1	KAE8788006.1	2	105	105	8.15	8.15	10,966.95	10,966.95	41.92	41.92	94	94	0.4	0.4
HvLTPd3/HtLTPd3	HORVU2Hr1G102170.1	KAE8781084.1	2	113	113	4.85	4.85	11,439.15	11,439.15	45.85	45.85	90.71	90.71	0.36	0.36
HvLTPd4/HtLTPd4	HORVU2Hr1G073730.1	KAE8786049.1	2	125	125	8.67	8.67	13,033.35	13,033.35	55.43	55.43	100	100	0.131	0.131
HvLTPd5/HtLTPd5	HORVU2Hr1G102050.1	KAE8787999.1	2	105	105	8.47	8.47	10,985.01	10,985.01	43.12	43.12	92.19	92.19	0.345	0.345
HvLTPd6/HtLTPd6	HORVU2Hr1G102100.1	KAE8788000.1	2	105	105	8.47	8.47	10,994.02	10,994.02	41.29	41.29	92.19	92.19	0.348	0.348
HvLTPd7/HtLTPd7	HORVU2Hr1G104430.1	KAE8799817.1	2	132	132	7.53	7.53	14,042.31	14,042.31	55.15	55.15	79.92	79.92	0.108	0.108
HvLTPd8/HtLTPd8	HORVU4Hr1G082600.1	KAE8799709.1	4	98	98	4.85	4.85	10,347.06	10,347.06	55.61	55.61	81.84	81.84	0.105	0.105
HvLTPd9/HtLTPd9	HORVU7Hr1G102030.1	KAE8770127.1	7	114	114	8.14	8.14	11,569.65	11,569.65	54.73	54.73	84.04	84.04	0.325	0.325
HvLTPd10/HtLTPd10	HORVU5Hr1G109100.1	KAE8808658.1	5	183	183	8.11	8.11	18,559.7	18,559.7	29.33	29.33	105.19	105.19	0.509	0.509
HvLTPd11/HtLTPd11	HORVU7Hr1G026570.1	KAE8795354.1	7	103	103	9.22	9.22	10,461.46	10,461.46	37.37	37.37	94.08	94.08	0.481	0.481
Type G															
HvLTPg1/HtLTPg1	HORVU1Hr1G009490.1	KAE8777605.1	1	177	177	7.48	7.48	16,847.26	16,847.26	44.5	44.5	86.21	86.21	0.482	0.482
HvLTPg2/HtLTPg2	HORVU2Hr1G098500.2	KAE8795728.1	2	189	189	6.06	6.06	19,086.3	19,086.3	65.61	65.61	90.9	90.9	0.439	0.439
HvLTPg3/HtLTPg3	HORVU4Hr1G071790.1	KAE8805373.1	4	194	194	5.52	5.52	18,793.44	18,793.44	67.86	67.86	80.26	80.26	0.389	0.389
HvLTPg4/HtLTPg4	HORVU5Hr1G109040.1	KAE8808654.1	5	197	197	8.66	8.66	19,981.15	19,981.15	44.98	44.98	86.4	86.4	0.335	0.335
HvLTPg5/HtLTPg5	HORVU5Hr1G076000.1	KAE8769277.1	5	190	190	4.41	4.41	19,076.74	19,076.74	68.97	68.97	76.26	76.26	0.295	0.295
HvLTPg6	MLOC_57612.1		5	176		8.47		17,033.39		52.61		81.7		0.372	
HvLTPg7/HtLTPg7	HORVU0Hr1G016430.1	KAE8793545.1	0	174	174	7.5	7.5	17,574.28	17,574.28	46.75	46.75	91.61	91.61	0.277	0.277
HvLTPg8/HtLTPg8	HORVU6Hr1G082080.1	KAE8808175.1	6	200	200	8.76	8.76	19,385.55	19,385.55	85.08	85.08	93.3	93.3	0.485	0.485

*CDS* coding sequence, *bp* base pair, *aa* amino acid, *MW* molecular weight, *Da* Dalton, *PI* isoelectric point

Based on the Mw of the mature proteins, plant nsLTPs can be classified into two main types: nsLTP1 (9 kDa) and nsLTP2 (7 kDa). Then, according to sequence similarity, Boutrot divided the nsLTPs into nine types (I, II, III, IV, V, VI, VII, VIII and IX) [4]. Recently, plant nsLTPs have been categorized into four major and several minor types (1, 2, C, D, E, F, G, H, J, K, X) by intron position, sequence identity and spacing between the cysteine residues in the 8CM, as well as post-translational modifications [41, 42]. Compared with the classification proposed by Edstam, 40 HvLTPs and 35 HtLTPs could be divided into four types, including 16/13(type 1), 5/5(type 2), 11/10(type D) and 8/7(type G) nsLTP genes (Table 1). As shown in Table 1, the molecular weights of HvLTPs and HtLTPs are usually between 9206.81 Da and 19,981.15 Da, and the molecular weight of Type 2 is smaller than that of the other types, with an average of 9 kDa. The molecular weight of the type G nsLTPs is relatively large and mostly between 16 and 19 kDa, with an average of 18 kDa. In the type G nsLTPs, the transcripts encode not only a C-terminal signal sequence, but also an N-terminal sequence, thus leading to a post-translational modification which a glycosylphosphatidylinositol (GPI)-anchor is added to the protein to attach the protein to the exterior side of the plasma membrane [43], leading to a much higher molecular weight of the type G nsLTPs than other types.

**Fig. 2:**
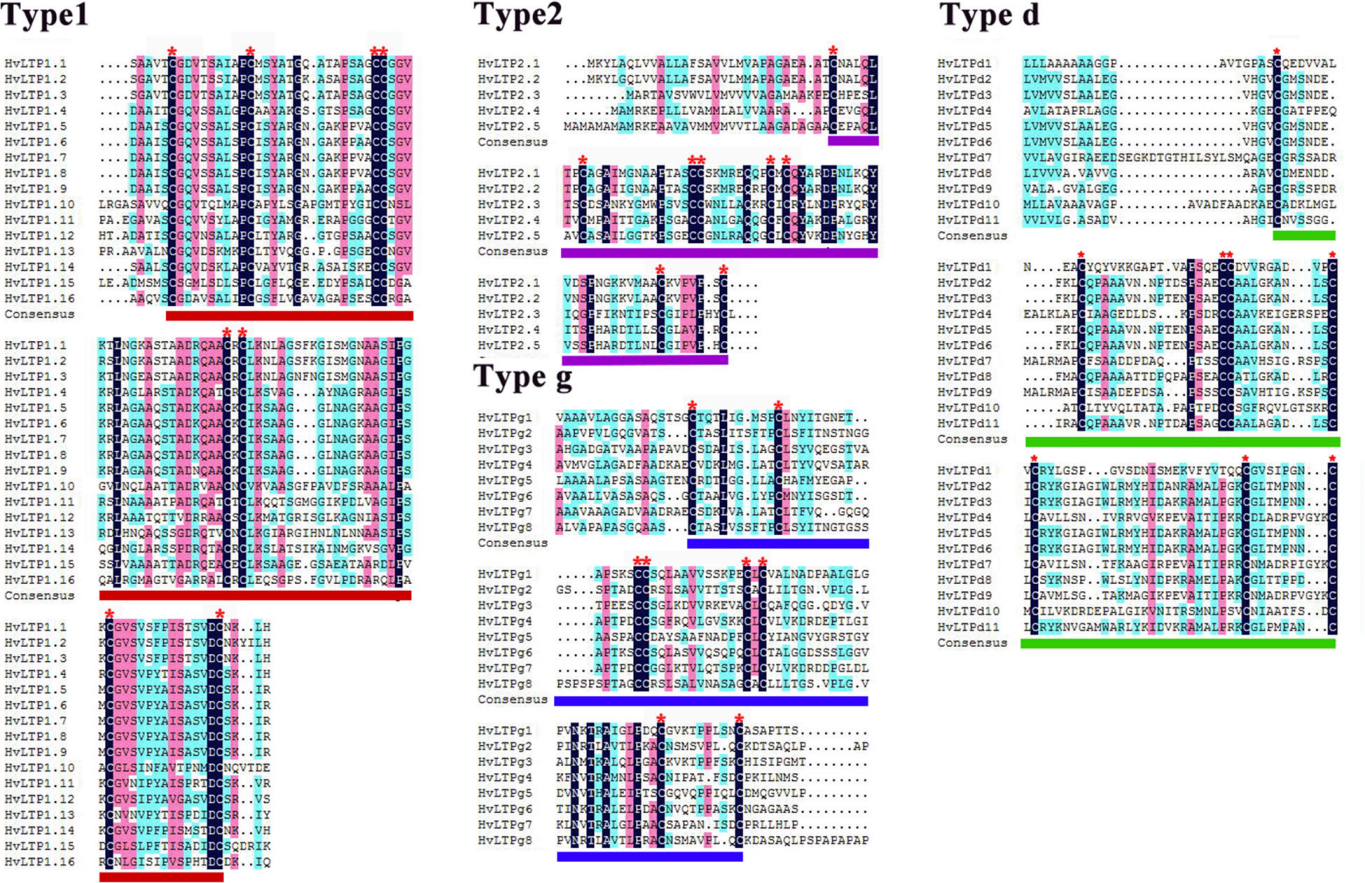
Multiple sequence alignment of the HvLTP and HtLTP 8CM domain sequences. The horizontal line represents the 8CM domain. * is Eight cyc in 8CM

The main characteristic of plant nsLTPs is the presence of 8CM motifs. To establish a specific 8CM consensus for each nsLTP type obtained, we conducted a multiple sequence alignment using the 8CMs from 40 HvLTPs and 35 HtLTPs. The amino acid sequence alignment of the 8CMs of HvLTPs and HtLTPs revealed a variable number of inter-cysteine amino acid residues. We also found that the alignment results are consistent with the classification results. For the CXC motif, most of the residues at the X position in type 1 nsLTP are only hydrophilic, while in type 2, D and G nsLTPs, the X position is usually occupied by hydrophobic residues (Fig. 2). These conserved hydrophobic or hydrophilic residues may play an important role in the biological function of HvLTPs and HtLTPs and are also consistent with their classification.

### Phylogenetic analysis, conserved motifs, and gene structure of the HvLTP and HtLTP families

**Fig. 3:**
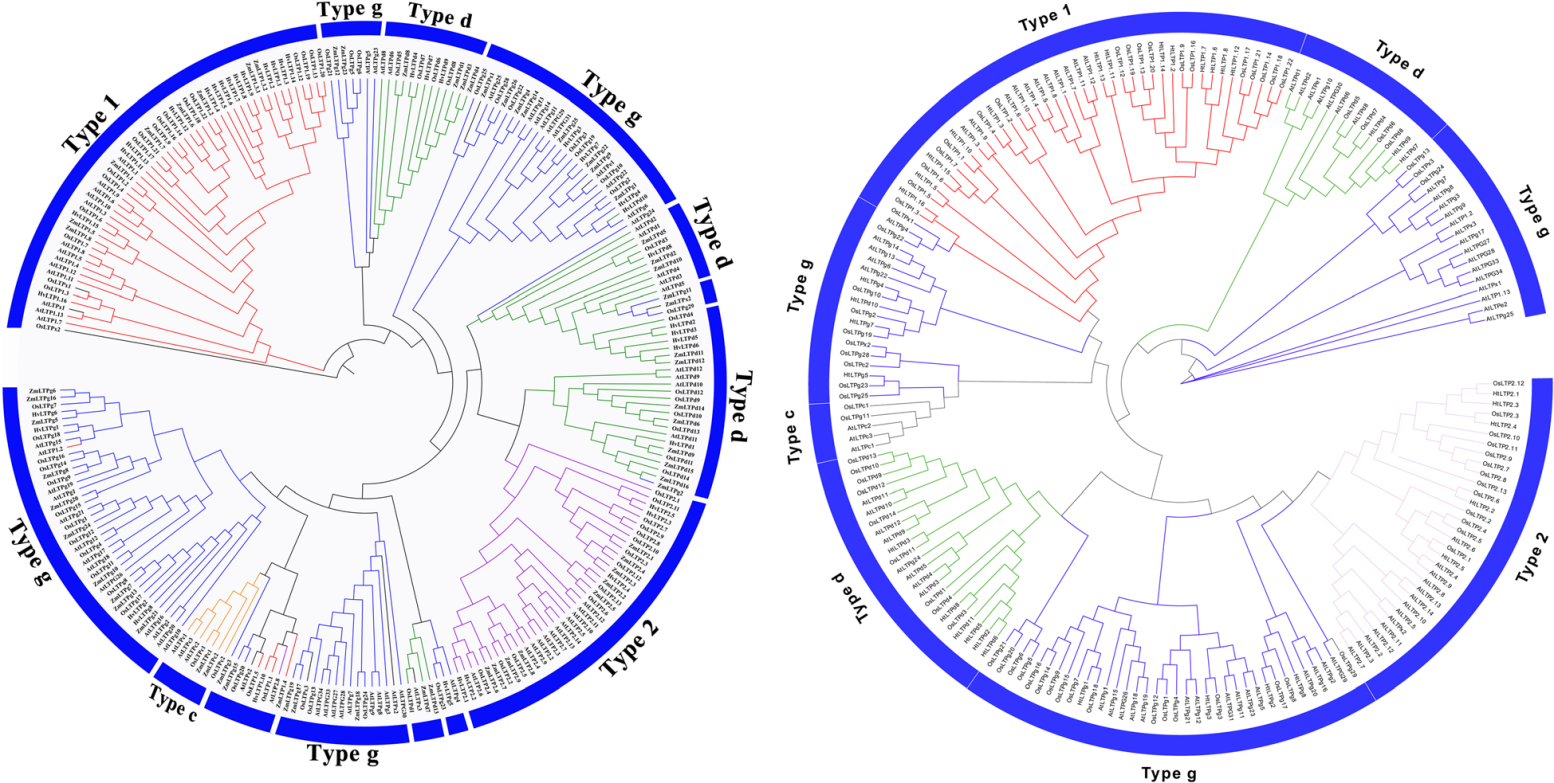
Unrooted phylogenetic tree representing the relationships of the nsLTP proteins of barley/Qingke, maize, rice, and *Arabidopsis thaliana*. The tree divided the HvLTP and HtLTP proteins into 4 types represented within the tree. The left/right phylogenetic tree was constructed from the nsLTP protein sequence of barley/Qingke, maize, rice, and *Arabidopsis thaliana*. The phylogenetic tree was derived using the neighbour-joining (NJ) method with 1000 bootstrap replications in MEGA-X

To analyse the evolutionary relationship, a phylogenetic tree of 293 nsLTPs from four species, including the HvLTPs and HtLTPs with maize, rice and Arabidopsis, was constructed. Comparing the previous classification data with phylogenetic analysis, it was found that the previous classification and phylogenetic analysis have the same type of nsLTPs; that is, the four groups in the HvLTP and HtLTP classification were consistent with the 1, 2, D and G types of the nsLTPs of the other three species, except for sporadic interlaces in types D and G (Fig. 3). The members of types 1 and 2 formed specific clades, indicating that these genes share a common ancestor in major nsLTP types.

**Fig. 4:**
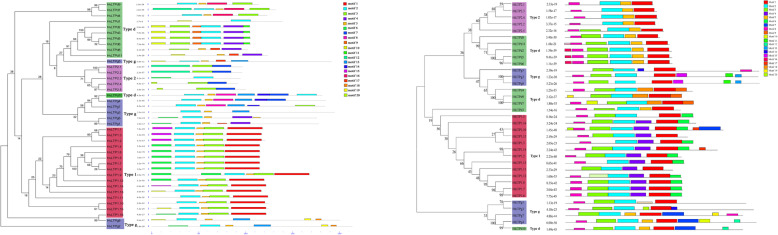
Schematic representation of twenty conserved motifs in the HvLTP (left) and HtLTP (right) gene families. Different coloured frames represent different protein motifs, and each motif has its own number

Based on the distribution of predicted motifs, 40 HvLTPs and 35 HtLTPs were categorized into four distinct subfamilies, which was consistent with the classification from the phylogenetic analysis (Fig. 4). Both the HvLTPs and HtLTPs have four different subfamilies. Type 1 has a similar motif, and the other types of motif structures are completely different. Common motif 2 was present in all HvLTPs and HtLTPs, except for six genes in type 2 (HvLTP2.1/HtLTP2.1, HvLTP2.2/HtLTP2.2, HvLTP2.4/HtLTP2.4 and HvLTP2.5/HtLTP2.5) and type D (HvLTPd8/HtLTPd8 and HvLTPd11/HtLTPd11). Special motifs appear in special types; for instance, motifs 1 and 15 are only present in Type 1, motifs 14 and 16 are only present in Type D, and motif 13 only exists in Type G HvLTPs and HtLTPs.

**Fig. 5:**
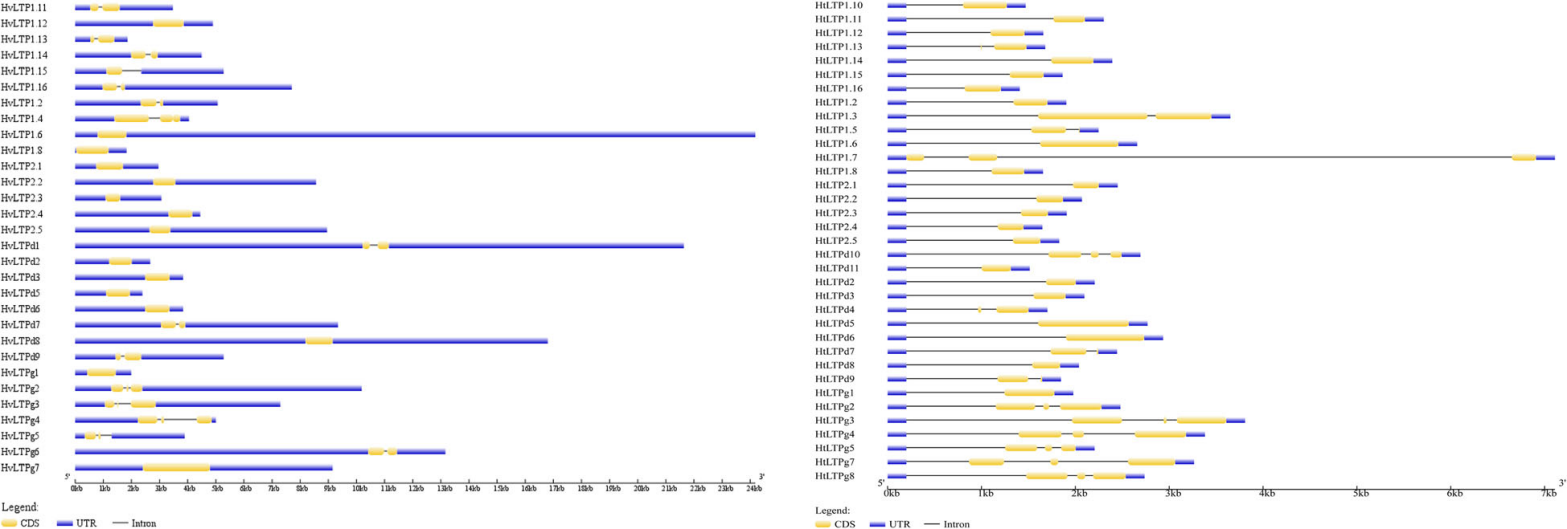
Exon–intron structure of the *HvLTP* (left) and *HtLTP* (right) genes. Exons, introns, and untranslated regions (UTRs) are indicated by yellow frames, grey lines, and blue frames at the bottom, respectively

As a type of evolutionary relic, the intron-exon arrangement carries the imprint of gene family evolution. The gene structures of the HvLTPs and HtLTPs were also investigated (Fig. 5). Investigation of the HvLTP and HtLTP gene structures revealed a low diversity distribution of intronic regions amid the exonic sequences. 40 HvLTP and 35 HtLTP genes were predicted to be interrupted by 0–2 introns positioned −9 to 104 bp downstream of the codon encoding the eighth cysteine in 8CM (Table 1). Additionally, it was interesting to find a similar exon/intron pattern in each group. For instance, the HvLTP and HtLTP genes in type 2 lack introns while the type G contains 2 introns. Except for HvLTPd1, HvLTPd7/HtLTPd7, and HvLTPd9/HtLTPd9, no introns were detected in the coding regions of type D genes.

### Chromosomal localization and gene duplication of *HvLTPs* and *HtLTPs*

**Fig. 6:**
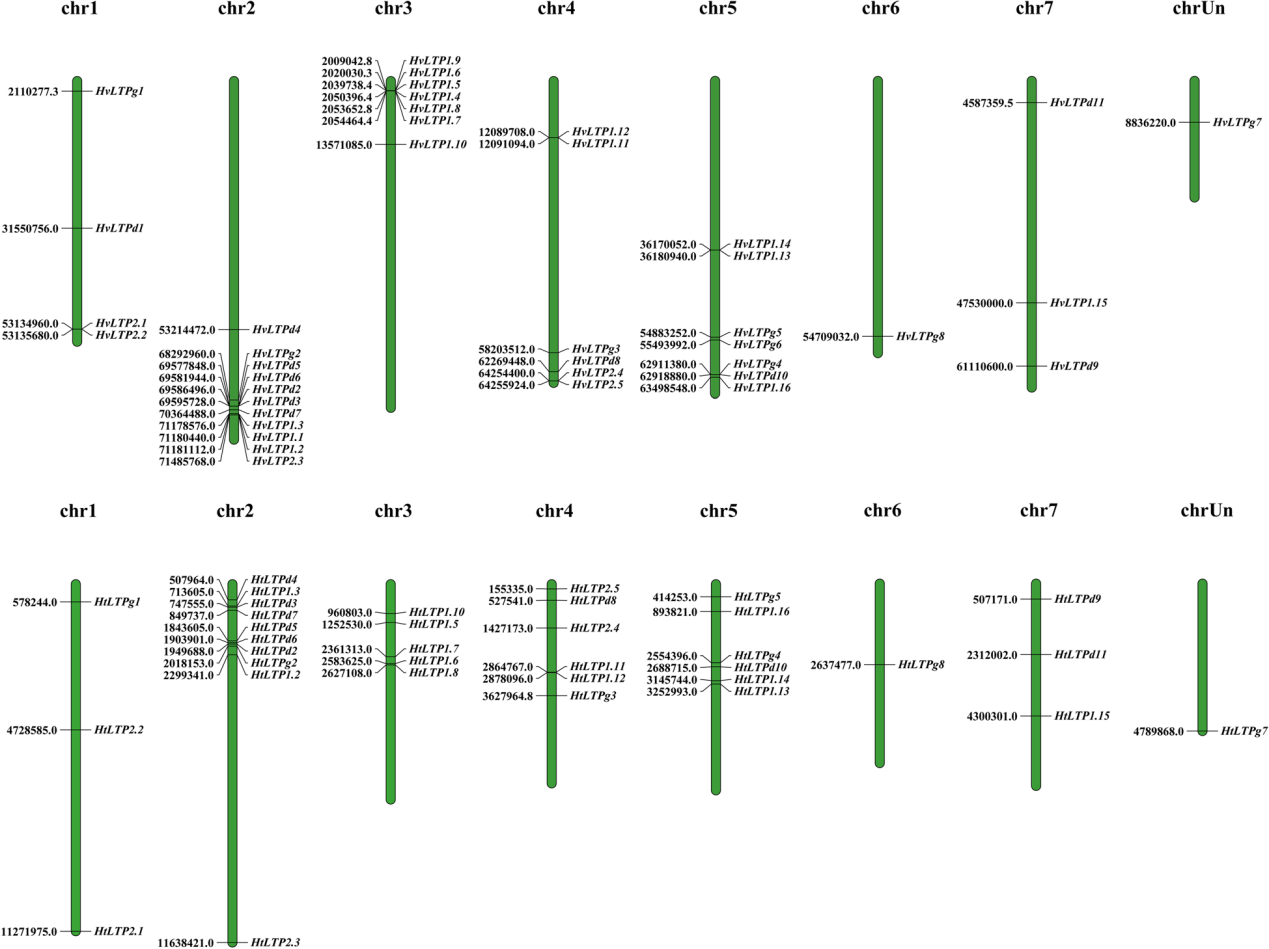
Chromosome localization of the *HvLTP* and *HtLTP* genes among 7 chromosomes. The left represents barley chromosomes, and the right represents Qingke chromosomes. Chromosomes are represented by cylinders, and the chromosome number is at the top of each chromosome. The location of nsLTPs is indicated on both sides of each chromosome.s

After determining the genomic location information of 40 HvLTP and 35 HtLTP genes, the genomic location information belonging to the 2012 IPK genes was not found for one of them (MLOC_57612.1). The Chromosomal localization results showed that 39 HvLTP and 35 HtLTP genes were unevenly distributed on 7 chromosomes of barley and Qingke, and there were obvious HvLTP and HtLTP gene clusters. The maximum number of HvLTP and HtLTP genes was contained on chromosome 2 (11), and the minimum number (1) was shown on chromosome 6 (Fig. 6).

Gene duplication is generally considered to be a major driving force in evolutionary innovation and leads to genomic complexity. In this study, six tandem repeats were identified in the barley genome (Table S2), which was consistent with obvious *HvLTP* gene clusters in the barley chromosome. Two significant clusters were found on chromosomes 2 and 3. In addition, one sister pair appeared to be generated from segmental duplication (Table S2, Figure S1A). Furthermore, we analysed the collinearity of *HvLTPs* with nsLTP genes in rice and wheat. Eleven out of 39 *HvLTP* genes had collinear genes with rice, while 22 *HvLTP* genes had syntenic members between barley and wheat (Figure S1B).

### Promoter and stress expression analysis of *HvLTPs* and *HtLTPs*

**Fig. 7:**
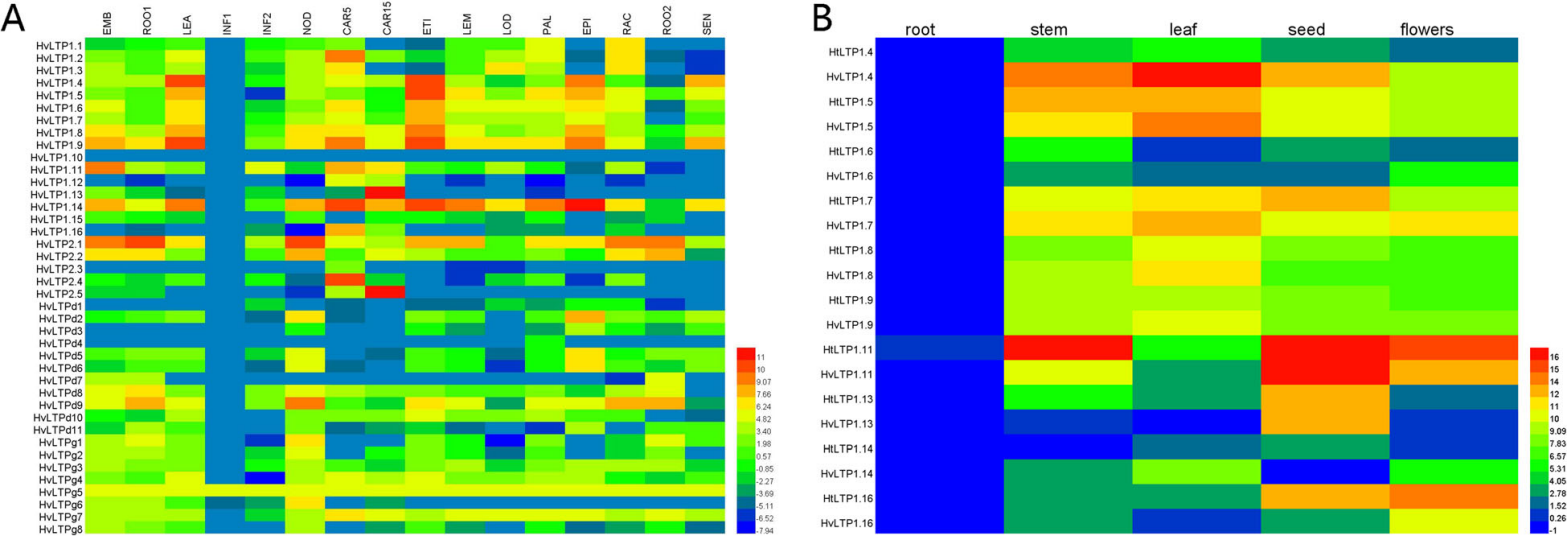
Tissue expression pattern in the *HvLTP* (left) and *HtLTP* (right) genes. On the left, the data were obtained from the BARLEX database at the Leibniz Institute of Plant Genetics and Crop Plant Research (IPK). X-axis: mRNA levels in 16 different tissues and life stages of barley. EMB: 4-day embryos; ROO1: roots from seedlings (10 cm shoot stage); LEA: shoots from seedlings (10 cm shoot stage); INF1: young developing inflorescences (5 mm); INF2: developing inflorescences (1–1.5 cm); NOD: developing tillers, 3rd internode (42 DAP); CAR5: developing grain (5 DAP); CAR15: developing grain (15 DAP); ETI: etiolated seedling, dark cond; LEM: Inflorescences, lemma (42 DAP); LOD: Inflorescences, lodicule (42 DAP); PAL: Dissected inflorescences, palea (42 DAP); EPI: Epidermal strips (28 DAP); RAC: Inflorescences, rachis (35 DAP); ROO2: Roots (28 DAP); and SEN: Senescing leaves (56 DAP). On the right, the data were obtained from the qRT-PCR results. X-axis: mRNA levels in 5 different tissues of Qingke. Different mRNA levels of each putative HvLTP and HtLTP are given as colour codes. Blue indicates a low expression level, and red indicates a high expression level

Plant nsLTPs display a complex tissue-specific and developmental expression patternand are mainly expressed in the tapetum, pericarp and epidermal cells of embryos, stems, leaves and roots [42–44]. According to on the RPKM values of each *HvLTP* gene published on the IPK website, an expression heatmap of 40 *HvLTP* genes in the 16 different tissues were mapped (Fig. 7A). The results showed that the *HvLTPs* had distinctly different expression patterns during different developmental stages as well as in different plant tissues. All *HvLTPs* were not expressed in INF1, and the expression level of *HvLTPs* in INF2 was also extremely low. Among the different *HvLTP* types, the relative expression levels of type 1 and type 2 genes were higher and more specific than those of type D and type G genes, except that the expression of HvLTP1.10 was lower in all tissues. Type 1 and type 2 *HvLTP* genes showed obvious tissue-specific expression patterns. For example, *HvLTP1.2* is mainly expressed in LEA. *HvLTP1.13* is specifically expressed in CAR15, and *HvLTP2.4* and *HvLTP2.5* are specifically highly expressed in CAR5 and CAR15, which indicates that these three genes might be involved in grain development. Given the high expression level of barley type 1 *HvLTPs* in the above transcriptome data, we simultaneously detected the tissue expression patterns of 10 type 1 barley and Qingke ns*LTP* genes in five different tissues by qRT-PCR (Fig. 7B). The results showed that the expression levels of type 1 *LTP* genes in barley and Qingke were very low in the roots, and the expression pattern of barley type 1 *HvLTPs* was basically consistent with that of transcriptome data. However, the expression patterns of 10 *nsLTP* genes were different in the barley and Qingke tissues. For example, the expression level of barley *LTP*1.4 was higher in the leaves, while that of Qingke *LTP*1.4 was lower in the leaves. The expression level of barley *LTP*1.11 was higher only in the seeds, and that of Qingke *LTP*1.11 was higher in the stems and flowers.

**Fig. 8:**
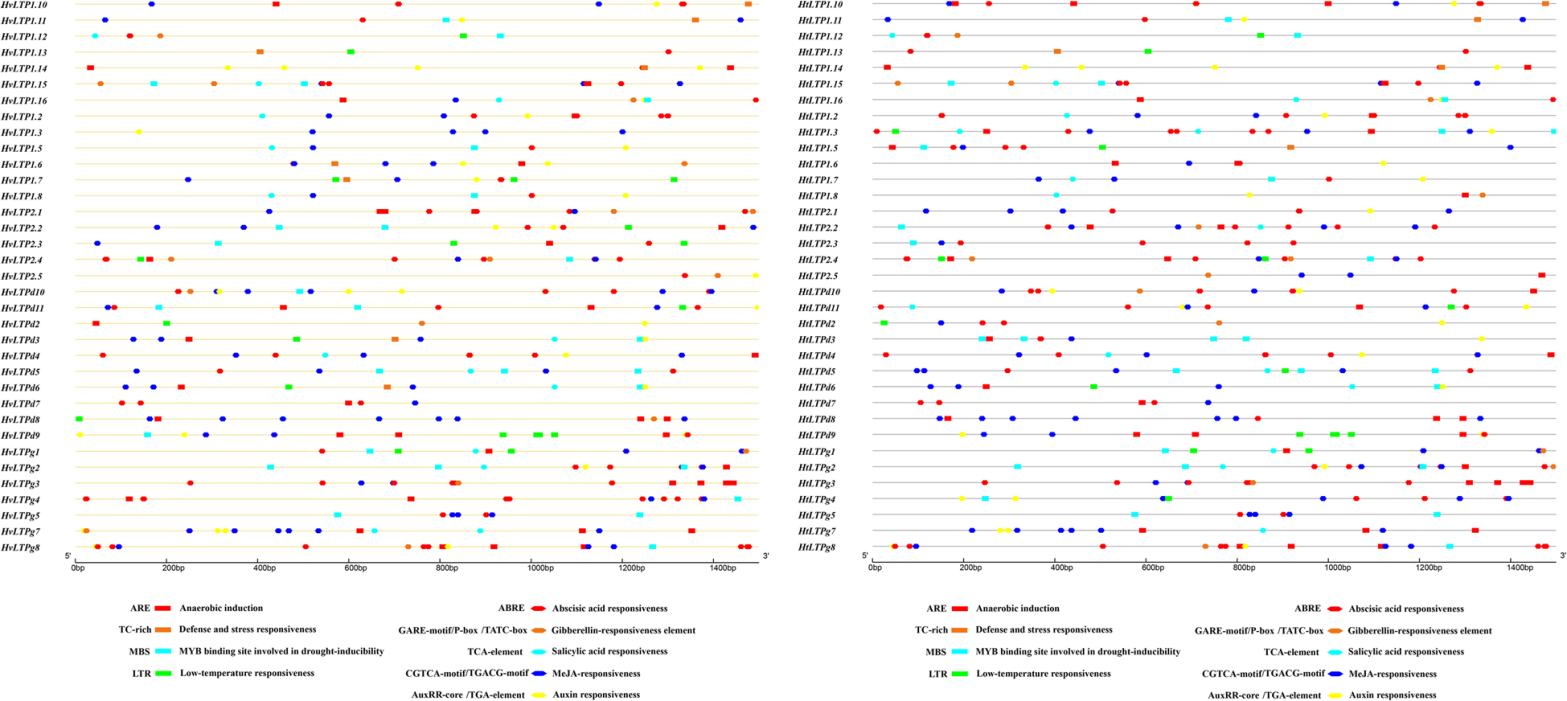
Prediction of cis-acting elements in the *HvLTP* (left) and *HtLTP* (right) promoters. Many cis-acting elements were detected in the promoter region of each *HvLTP* and *HtLTP* gene; and different colours and shapes represent different promoter elements

An in silico analysis of the 1.5 kb upstream region (starting from the translation initiation site) of 35 *HvLTP* and *HtLTP* genes revealed various regulatory elements associated with development and abiotic or biotic stress signalling (Fig. 8.). In addition to the TATA-box and CAAT-box, the A-box is the most common type of cis-elements in the *HvLTP* and *HtLTP* genes. In this study, we found that the cis-elements of the *HvLTP* and *HtLTP* genes included stress response elements (ARE, MBS, MYB, LTR, TC-rich motif, and DRE), hormone-related elements (ABRE, TGACG-element, CGTCA-motif, TGA-element, TCA-element, GARE-motif, P-box, TATC-box, AuxRR-core, ERE, and Wbox), indicating that *HvLTP* and *HtLTP* are involved in stress response and hormone signalling. Additionally, the regulatory element involved in light responsiveness also appears to be enriched in the *HvLTP* and *HtLTP* genes, including G-Box, ACE, Box 4, GT1-motif, Sp1, TCT-motif, GATA-motif, I-box, AE-box, Box II, and TCCC-motif. Some *HvLTP* and *HtLTP* genes have specific developmental response elements such as zein metabolism regulation (O_2_-site), meristem expression (CAT-box), and seed-specific regulatory elements (RY-elements). A comparison of the 35 gene promoters of *HvLTPs* and *HtLTPs* showed that only 6 genes had the same cis-element organization, indicating that there were more stress response- and hormone-related elements in Qingke. There are both similarities and differences between *HvLTPs* and *HtLTPs*. Similarly, all have a similar number of stress response elements, most of which have AREs (essential for the anaerobic induction), LTRs (involved in low-temperature responsiveness) and MBSs (MYB binding sites involved in drought-inducibility). For nsLTP, MBS has been previously reported as a target for MYB transcription factor to modulate plant tolerance to freezing and drought stress [45], suggesting that these *HvLTPs* and *HtLTPs* containing MBS or MYB elements may participate in abiotic stress signalling of MYB. The difference is that the number of hormone response elements and light responsive elements in Qingke was higher than in barley, and ABRE and G-box are the most important and different hormone and light-responsive regulatory elements in *HvLTP* and *HtLTP* genes, which may mean that Qingke may rely on the abscisic acid hormone pathway and G-box related light-response pathway to adapt to more severe plateau environment [39].

**Fig. 9:**
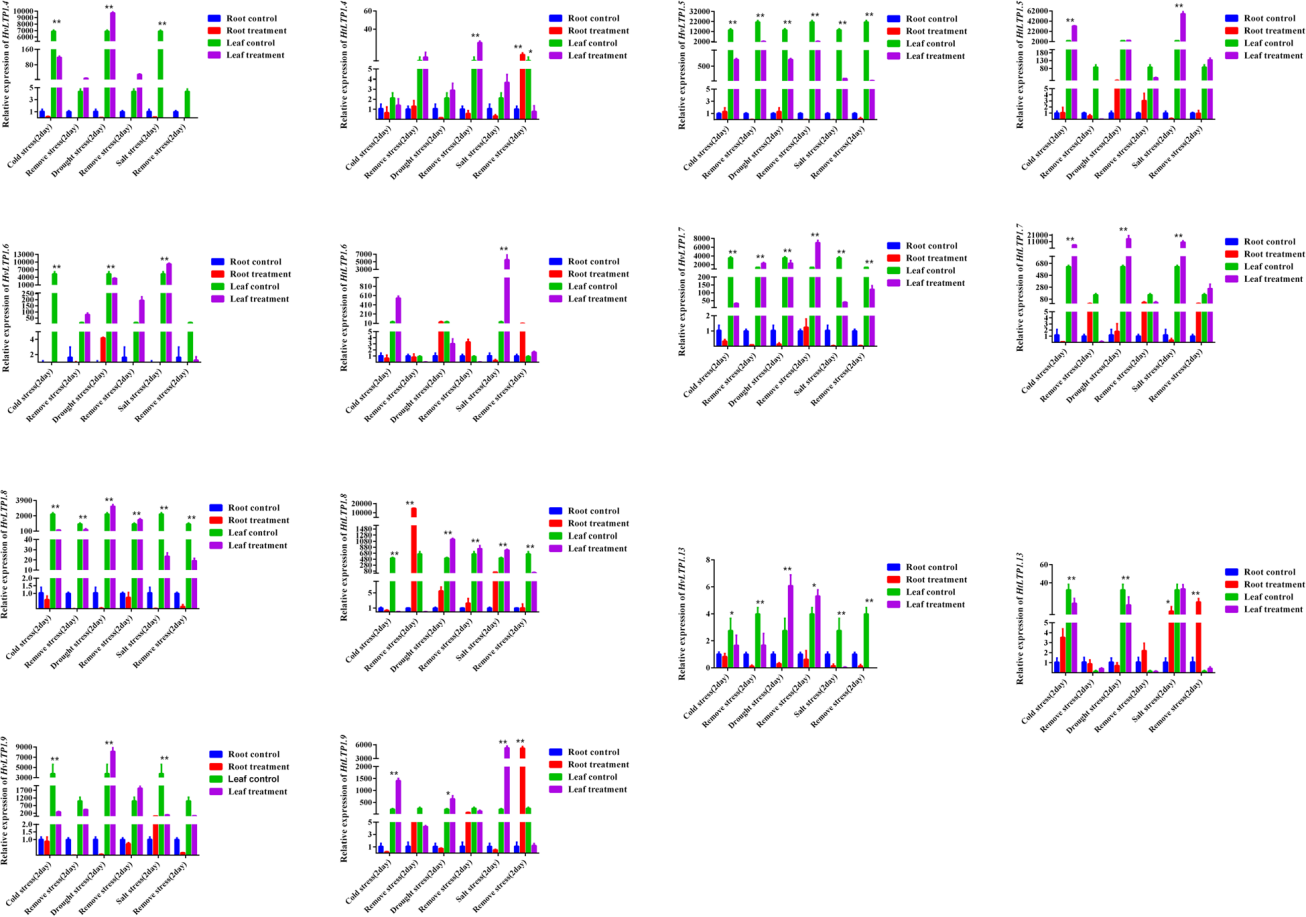
Expression profiles of 7 pairs of selected *HvLTP1* and *HtLTP1* genes in response to various abiotic stress treatments. Data were normalized to the actin gene, and vertical bars indicate the standard deviation. The relative expression levels of the *HvLTP1* and *HtLTP1* genes in plants grown under different abiotic stresses measured by qRT–PCR. The mean (± SE) expression values were calculated from three independent biological replicates and three technical replicates. (**P < 0.01; *P < 0.05; ns, *P* > 0.05)

qRT-PCR was performed further to explore the expression patterns of 16 *HvLTP* and 7 *HtLTP* genes in the root and leaf tissue under abiotic stress, which means that barley seedlings were treated for 2 days with drought, cold and salt stress, and then recovered for 2 days (Fig. 9, Figure S2). Similar to spatiotemporal tissue expression of *HvLTPs*, the expression level of Type 1 genes is higher than that of other types in *HvLTPs* under abiotic stress; therefore, the expression of type 1 genes of *HtLTP* genes were also assessed by qPCR assay. The seven Type 1 *HvLTP* and *HtLTP* genes had significant responses in the leaves under cold, drought and salt stress, but no or low responses in the roots, which was consistent with the low expression in the root in the IPK database (Fig. 9). The expression level of all barley Type 1 *HvLTP* genes was decreased under cold stress, while four *HtLTP* genes were upregulated in Qingke. The 4 Type 1 *HvLTP* and *HtLTP* genes were upregulated in barley and Qingke under drought stress, but two of them were different. For example, the expression of *HvLTP* 1.7 was downregulated in barley, but *HtLTP* 1.7 was upregulated in Qingke; and the expression of *HvLTP* 1.3 was upregulated in barley, but *HtLTP* 1.3 was downregulated in Qingke. Only one Type 1 *HvLTP* gene was upregulated in barley under salt stress, while six Type 1 *HtLTP* genes were upregulated in Qingke. In general, the expression patterns of seven Type 1 *HvLTP* and *HtLTP* genes in barley and Qingke under abiotic stress were different. After abiotic stress was removed, the change trend of the type 1 gene was also different between barley and Qingke. For example, there were 3 genes in barley while there were 6 genes with opposite change trend in Qingke under cold stress; there were 5 genes in barley but 4 genes with the same change trend in Qingke under drought stress; and there were 6 genes in barley but only 3 genes with the same change trend in Qingke under salt stress. At the same time, there were also differences in genes with opposite trends; for example, the *HvLTP* 1.4 and *HvLTP* 1.6 genes were downregulated first and then upregulated under cold stress in barley, while *HtLTP* 1.4 and *HtLTP* 1.6 were upregulated first and then downregulated in Qingke.

Unlike type 1, two genes (*HvLTP2.1/HvLTP2.2*) of type 2 responded significantly to drought, cold and salt stresses in the roots and *HvLTP2.1* responded significantly to salt stress in the leaves. The two genes showed the same change trends in the roots under the removal of abiotic stresses basically. For example, the two genes were upregulated continuously under cold stress and after the removal of cold stress, but they were downregulated under drought stress and upregulated after the removal of drought stress; moreover, slightly different were observed under salt stress. *HvLTP2.1* was upregulated continuously with and without salt stress, while the *HvLTP2.2* gene changed in opposite directions, i.e., it was downregulated first and then upregulated. Type D genes were downregulated under cold and drought stress, while most type D genes were upregulated under salt stress in the roots. The *HvLTPd2* and *HvLTPd5* genes also showed significant effects and were upregulated in the leaves under drought and salt stress. After the removal of abiotic stress, the overall fluctuation of type D genes was the opposite, but the *HvLTPd7* gene showed the same trend. In type G, the response of *HvLTPg1* in the roots was upregulated significantly under cold stress, while the response in the leaves was upregulated significantly under drought and salt stress. *HvLTPg2* was downregulated significantly in roots and leaves under the three abiotic stresses. Only *HvLTPg5* was upregulated significantly in leaves under cold stress. In general, the type G genes were not affected due to low expression after removal of abiotic stress, and only some genes had the opposite changing trends, including the *HvLTPg1* genes in roots and *HvLTPg2* genes in roots and leaves under drought stress; and the *HvLTPg*5 gene in leaves under the three abiotic stresses (Figure S2).

## Discussion

Plant nsLTPs are a large transporter family composed of 79 members in Arabidopsis, 77 in rice, 63 in maize, 58 in sorghum, 63 in cabbage and 156 in wheat, all of which are classified as different types [4, 17, 38, 39, 46]. In 2018, the wheat nsLTP family was comprehensively analysed again, and 461 putative TaLTPs were identified from the whole wheat genome [47]. Edstam provided comprehensive information about the categorization of the nsLTP gene family based on phylogenetic clustering and facilitated further functional analysis [41]. In this study, 40 and 35 nsLTPs were identified in the barley and Qingke genomes and classified into 4 subfamilies (Type 1, 2, D and G) following Edstam’s classification (Table 1, Fig. 2). In addition, the number and classification of HvLTPs were also compared with those in Arabidopsis, rice, maize and cabbage (Table 2). The results showed that the total number of nsLTP gene families in barley and Qingke was less than that in other species. Moreover, type E is unique to dicotyledons, while HvLTPs and HtLTPs are also deficient in type C and type X (Table 2). Meanwhile, the proportion of nsLTPs in each subfamily indicated that type G seemed to have contracted in barley, but no expansion was observed. Therefore, the decrease in nsLTP gene number in barley may be due to the loss of individual types and the reduction of individual populations. In addition, this number may be due to the inhibition of gene expansion. Zhang (2019) showed that 70 barley nsLTP genes were identified by keyword searching in Phytozome v12.1.6 and divided into five types (1, 2, C, D, and G) [40], which indicates that previous research results of this barley nsLTP gene family expansion are different from ours. Compared with Zhang’s research, our research collected more comprehensive data through a BLASTP search, HMM search and keyword search of the barley genomes, including in the IPK, Ensembl, NCBI and Phytozome databases, and obtained 160 potential HvLTP genes. Referring to previous screening methods [17], the screening method was also more stringent and finally identified 40 barley HvLTP genes; therefore, our results were more accurate, comprehensive and reliable.

**Table 2:** Numbers of nsLTP genes in different species

species	Total number of members	Type 1	Type 2	Type C	Type D	Type E	Type G	Type X
*Hordeum vulgare*	40	16	5	0	11	0	8	0
*Qingke*	35	13	5	0	10	0	7	0
*Arabidopsis thaliana*	79	13	13	3	12	2	33	3
*Oryza sativa*	77	18	13	2	14	0	27	3
*Zea mays*	63	8	9	2	15	0	26	3
*Brassica oleracea var. capitata*	89	19	12	1	18	2	28	9

The role of gene duplication in the origin of evolutionary novelty and complexity has long been recognized [48]. In our study, we found one segmental duplication and six tandem duplication events in the barley nsLTP gene family (Table S2; Figure S1). These results suggest that gene expansion is inhibited overall and the evolution of the barley nsLTP gene family not only involves gene retention but also gene loss and mutation. The retention and loss of genes may be associated with related functions during plant evolution [49]. In addition, these paralogous duplicated genes may retain some essential functions in subsequent evolution. For example, five pairs of tandem duplication genes (HvLTP1.1/HvLTP1.2/HvLTP1.3, HvLTP1.5/HvLTP1.7/HvLTP1.8, HvLTP1.6/HvLTP1.9, HvLTP2.1/HvLTP2.2, HvLTPd2/HvLTPd5/HvLTPd6) shared similar expression profiles. However, one pair of duplicated genes (HvLTP2.4/HvLTP2.5) showed significant divergence in expression. At the same time, another pair of segmental duplication genes (HvLTP1.12/HvLTP1.13) also showed different expression patterns. The differential expression patterns of these duplicated genes in barley indicated that these genes may be functionalized after duplication events during the evolutionary process, leading to significant variation in gene regulation [50, 51].

Sufficient evidences has been obtained to show that nsLTPs are involved in various types of stress resistance, including resistance to phytopathogens, freezing, drought and salt [42, 52]. In our study, the stress-dependent cis-elements in the promoter regions of the *HvLTP* and *HtLTP* genes were analysed. The results showed that the promoters of the *HvLTP* and *HtLTP* genes contained stress response elements (STRE, DRE, MBS, MYB, TC-rich repeats) and hormone-related elements (ARE, LTR, ABRE, ERE, TCA-element, TGA-element, TGACG-element, CGTCA-motif, and W box), indicating that the *HvLTPs* and *HtLTPs* are involved in the stress response. Meanwhile, the expression patterns of 17 HvLTP genes in response to abiotic stresses also demonstrated their correlation with abiotic stresses. In general, the response of these genes in the root was weaker than that in the leaf. Under different abiotic stresses, the response levels of different type members were inconsistent. For example, the response of *HvLTP* type 1 was significantly higher than that of the other subfamilies. The *blt4* gene of barley is a low-temperature response gene with different responses to drought, pathogen attack, and abscisic acid (ABA) [28]. Previous studies have shown that *blt4* belongs to the barley nsLTPs located on chromosome 3 and can act as a regulatory protein to stabilize plasma membrane activity and resist low-temperature injury [12]. Interestingly, blt4 genes belong to the barley nsLTP type 1, and some of them appear in tandem duplication events, such as blt4.3 (HvLTP1.8)/blt4.9 (HvLTP1.7) and blt4.1 (HvLTP1.6) (Table S2, Fig. 6). The expression patterns of these tandem duplication genes were similar, indicating that these genes may retain the same function and coordinate the regulation of the stress response through the tandem repeat of gene clusters.

Different promoter cis-elements and their epigenetic changes have been reported to affect gene regulation, thereby resulting in different gene expression levels and further affecting adaptation to the environment, including altitude changes [53–55]. We also compared the nsLTP gene promoter and stress expression pattern between barley and Qingke and found that the nsLTP gene sequences between barley and Qingke were basically the same, but the promoter and stress expression pattern were different. Moreover, the expression of nsLTP gene was directly related to its promoter, which indicated that the stress expression pattern of nsLTP gene was changed by its promoter. Combined with the analysis of upstream cis-elements of *HvLTPs* and *HtLTP*s and their expression patterns under abiotic stress, it was found that a large number of *HvLTP* and *HtLTP* genes may change their regulatory modes due to different upstream cis-elements and cause different abiotic stress responses. Generally, there is little difference between them in terms of the *HvLTP* and *HtLTP* genes themselves, including the gene structure, conserved motifs, phylogenetic analysis and classification. The main difference between them lies in its promoter regulatory elements, which may lead to the difference of expression patterns between *HvLTP* and *HtLTP* genes, thus leading to the adaptability of Qingke to extreme plateau climate.

## Conclusions

In summary, 40 HvLTPs and 35 Qingke nsLTPs were identified in barley and Qingke in this study. A comprehensive study of HvLTPs and HtLTPs will reveal important features of the nsLTP gene family, such as gene structure, evolution, chromosome distribution, conserved motifs, segmental and tandem duplication, upstream cis-elements, and stress expression patterns. The study results could be considered a useful source for future nsLTP gene research in either barley or Qingke or for comparisons of different plant species. This study will help to provide a foundation for future research on the molecular mechanisms of barley and Qingke stress adaption.

## Materials and methods

### Plant materials, growth conditions and abiotic stress treatment

The barley variety ‘Morex’ and Qingke variety ‘Dulihuang’ were used as the plant material in this study. The seeds were surface sterilized with 10% H_2_O_2_ (v/v) for 10 min and rinsed with deionized water for several times. Then, the soaked seeds were kept at 25 °C for 48 h in darkness to germinate in a light growth chamber. The germinating seeds were planted in 1.5 L pots, filled with Hoagland’s nutrient solution and were grown in a greenhouse at 22 °C with a photoperiod of 16 h (12,000 lx) and a dark period of 8 h. Seedlings were grown to maturity (14 days of germination) under normal conditions and then treated under different abiotic stress conditions including drought, cold and salt. For salt stress, 200 mM NaCl was added to the nutrient solution for 48 h; for drought stress, 18% polyethylene glycol was added to the nutrient solution for 48 h; and for cold stress, the seedlings were kept at 4°Cfor 48 h. Then, tissue samples composed of leaves and roots from every stress treatment were collected once. After 48 h, the seedlings were transferred to nutrient solution without stress and cultured for 48 h in the growth chamber. Then, the tissue samples were taken again. Then, the plant materials were collected and immediately frozen in liquid nitrogen for RNA extraction. All samples were replicated three times.

### Identification of putative nsLTP genes in the barley and Qingke genomes

All known nsLTP amino acid sequences from Arabidopsis (*A. thaliana*), maize (*Zea mays* L), cabbage (*Brassica rapa* L) and rice (*O. sativ*a) were used as queries (Table S1) by searching against the barley database using the BLASTP (IPK) program with the default parameters (http://webblast.ipk-gatersleben.de/barley_ibsc/). Simultaneously, an HMM search was performed on the barley genome release-41 from Ensembl Genomes (http://ensemblgenomes.org/), and amino acid sequences containing the domain PF00234 (Tryp alpha amyl domain, plant lipid transfer/seed storage/trypsin-alpha amylase inhibitor) were obtained. In addition, the proteins associated with nsLTP were searched by keywords from the NCBI (https://www.ncbi.nlm.nih.gov/), IPK (http://apex.ipk-gatersleben.de/apex) and Phytozome (https://phytozome.jgi.doe.gov/pz/portal.html) databases. The results from the BLASTP, HMM and keyword searches were combined to remove redundant sequences. To increase the probability of detecting putative nsLTPs in barley, all barley protein sequences were downloaded from the NCBI and IPK databases and submitted to the BatchWeb CD-Search Tool (http://www.ncbi.nlm.nih.gov/Structure/cdd/wrpsb.cgi) to verify the presence of nsLTP domain cl07890(PF00234 belong to cl07890). Pfam (http://pfam.sanger.uk/) validation was then performed using the domain PF00234. Then, the deduced protein sequences of candidate nsLTPs were manually examined to determine whether they harboured the 8CMs (C … C … CC … CXC … C … C), and proteins lacking the essential cysteine residues were removed. Subsequently, the proteins without NSSs (N-terminal signal sequence prediction, http://www.cbs.dtu.dk/services/SignalP, checked by the PROTTER http://wlab.ethz.ch/protter/start/) were also removed, and the remaining C-terminal glycosylphosphatidylinositol (GPI) anchors (GPI anchor signal prediction, http://mendel.imp.ac.at/gpi/plant_server.html and http://psort.hgc.jp/form.html) remained. Subsequently, putative proline-rich or hybrid proline-rich proteins, which are characterized by a high proportion of proline, histidine and glycine residues in the sequence located between the NSS and the 8CM, were excluded from further analyses. The protein sequences of At2S1-At2S4 and RAT1 were then BLAST-searched against the rest of the candidate nsLTP proteins to exclude possible inhibitors and cereal storage proteins. Proteins with more than 120 amino acids at maturity were also discarded, and the final remaining amino acid was identified as the target amino acid sequence, named HvLTPs (the nsLTPs in barley). The entire screening process was undertaken strictly according to Fig. 1.

The Qingke (Tibetan hulless barlay) genome was downloaded from the NCBI (https://ftp.ncbi.nlm.nih.gov/genomes/genbank/plant/Hordeum_vulgare/all_assembly_versions/GCA_004114815.1_Hulless_Barley_ass.V2/), and putative Qingke nsLTPs Orthologues were identified using HvLTP sequences by local BLAST and named HtLTPs (nsLTPs in Qingke).

### Multiple sequence alignment and classification

The amino acid sequences of the putative 40 HvLTPs and 35 HtLTPs were downloaded and multiple alignment of the 8CM part of these sequences was then conducted and manually edited using the DNAMAN program. Additionally, the amino acid sequences were submited to the online site Compute pI/Mw tool (http://web.expasy.org/compute_pi/) to calculate the isoelectric point and molecular weight. Sub-cellular localization of these genes was predicted by the Plant-mPLoc online service (http://www.csbio.sjtu.edu.cn/bioinf/plant-multi/). The three-dimensional structures of all putative HvLTPs and HtLTPs were also predicted by SWISS-MODEL (https://swissmodel.expasy.org).

The HvLTPs and HtLTPs can be divided into four major and several minor types according to sequence identity, spacing between the Cys residues and intron position in the 8CM, and the post-translational modifications based on the presence of a GPI modification site. In the second round of classification, the HvLTPs and HtLTPs were sorted based on the identity matrix calculated from the multiple sequence alignments [41].

### Phylogenetic construction

The nsLTP amino acid sequences of Arabidopsis, maize, cabbage and rice were downloaded from the TAIR (http://www.Arabidopsis.org/), gramene (http://ensembl.gramene.org/Zea_mays/Info/Index), BRAD (http://brassicadb.org/brad/index.php) and RGAP (http://rice.plantbiology.msu.edu/) databases, respectively. Multiple alignments of the mature proteins were carried out and phylogenetic tree was built using MEGA 5.0, with the neighbour-joining (NJ) method and 1000 bootstrap replications. After that, the results were imported to the iTOL (https://itol.embl.de/) online service output picture.

### Protein motif and gene structure analysis

The predicted barley and Qingke nsLTP protein sequences were submitted to the online MEME (http://meme-suite.org/tools/meme) to identify 20 distinct conserved motifs in the nsLTPs. The following parameters were used: repetitions are arbitrary, maximum number of bases is 10, and optimal base widths are limited to between 6 and 50 residues.

The prediction analysis of gene structure was carried out using GSDS (http://gsds.cbi.pku.edu.cn/) using the DNA and cDNA sequences of each predictive HvLTP and HtLTP gene from the barley and Qingke genomes.

### Chromosomal mapping and gene duplications

The chromosome location information of the nsLTPs was searched in the barley and Qingke genome databases and MapInspect software was used to generate chromosomal distribution images.

Gene duplication was investigated following the method described by Kong *et al* [56]. The MCScanx and Circos programs were used to retrieve and map the collinearity between different plant genomes.

### Promoter analysis of *HvLTPs* and *HtLTPs*

To investigate the cis-elements in promoter sequences of barley and Qingke nsLTP genes, the upstream sequences (~1500 bp) of each identified *HvLTP* and *HtLTP* were retrieved from the barley and Qingke genomes using a Perl script. The PlantCARE website (http://bioinformatics.psb.ugent.be/webtools/plantcare/ html/) was used to identify cis-elements in the promoters.

### Tissue expression profile analysis of *HvLTPs*

The publicly available Barley RNA-Seq datasets were downloaded from the IPK database, and the value of fragments per kilobase of transcript per million fragments mapped (FPKM) of these genes was used visualize the heat map using HEMI software and then used to analyse the barley tissue expression profiles of the identified *HvLTP* genes.

### RNA isolation and quantitative real-time PCR

Total RNA was extracted using the Plant RNA isolation kit (Takara, Shiga-ken, Japan) following the manufacturer’s instructions. The RNA quality was checked using a 1.0% (w/v) agarose gel stained with ethidium bromide (EB), and the RNA samples were inspected for quality and quantity using a NanoDrop® spectrophotometer and gel imager analysis. First-strand cDNA was synthesized from DNase-treated RNA with a PrimerScript 1st Strand cDNA synthesis kit (TIANGEN, Beijing, China). *HvLTP* and *HtLTP* gene-specific primers were designed based on their coding sequences (CDSs) using an online tool in NCBI (https: //www.ncbi.nlm.nih.gov/tools/primer-blast) and then synthesized commercially (Shenggong, Shanghai, China) (Table S3). qRT-PCR was performed with SYBR GREENI and the CFX96 Real-time System (Bio-Rad, France) by strictly following the manufacturer’s instructions. The thermal cycling conditions were as follows: 50 °C for 2 min, 95 °C for 10 min, and 40 cycles of 95 °C for 15 s. The relative transcription levels were calculated using the 2^−ΔΔ^ CT method, and three technical replicates were performed for each sample.

## Supplementary Information

**Additional file 1: Table S1.** Query genes for the barley nsLTP gene family screening.

**Additional file 2: Table S2.** Gene duplication events for the *HvLTP* genes in each barley chromosome.

**Additional file 3: Table S3.** Quantitative PCR primers for the *HvLTP* (*HtLTP)* genes.

**Additional file 4: Figure S1.** Collinear analysis of the inter-species (A) and intra-species (B) comparison of *HvLTP* genes.

**Additional file 5: Figure S2.** Expression analysis of type 2, type D and type G *HvLTP* genes.

## Data Availability

All data generated or analysed during this study are included in this article and its additional files. The genome data of *Hordeum vulgare* was downloaded from https://webblast.ipk-gatersleben.de/downloads/barley_pangenome/Morex/ and http://plants.ensembl.org/Hordeum_vulgare/Info/Index [29, 30], the genome data of *Qingke* were downloaded from https://ftp.ncbi.nlm.nih.gov/genomes/genbank/plant/Hordeum_vulgare/all_assembly_versions/GCA_004114815.1_Hulless_Barley_ass.V2/ and http://www.ibgs.zju.edu.cn/ZJU_barleygenome.htm [22, 31], All the RNA-Seq data are available at IPK from https://apex.ipk-gatersleben.de/apex/f?p=284:57.

## Abbreviations

nsLTP: Plant non-specific lipid transfer proteins
8CM(ECM): Eight cysteine residues motifs
IPK: Leibniz Institute of Plant Genetics and Crop Plant Research
NSS: N-terminal signal sequences
GPI: C-terminal glycosylphosphatidylinositol anchors
qRT-PCR: Quantitative real-time PCR
